# A reflection on paradigmatic tensions within the FDA advisory committee for MDMA-assisted therapy

**DOI:** 10.1177/02698811241309611

**Published:** 2024-12-26

**Authors:** Leor Roseman

**Affiliations:** Department of Psychology, University of Exeter, Exeter, UK

**Keywords:** Paradigm shift, psychedelics, psychopharmacology, psychiatry, set and setting

## Abstract

The recent rejection of 3,4-methylenedioxymethamphetamine (MDMA)-assisted therapy by the U.S. Food and Drug Administration (FDA) is a dramatic moment in the re-emergence of psychedelic research. In this perspective, I argue that it represents a case study for paradigmatic tensions within psychopharmacology. The regulatory system is still influenced by a paradigm that sees the therapeutic effects of drugs as primarily biological, and context is noise to control for. An emergent paradigm considers the therapeutic effects of drugs as interactive with context. Psychedelics are the anomaly that questions the dominant paradigm, mainly due to the determination of psychedelic researchers that the medicines are drugs with psychotherapy. While some of the critique offered by the FDA towards MAPS/Lykos’s studies is crucial, much of it is related to the experiential and psychotherapeutic elements – which the FDA claims not to regulate. This leads to some paradoxes within the regulatory procedure, which hint at a need for a shift in how psychedelic-assisted therapy is regulated and researched. Both regulators and researchers will need to find ways to accommodate each other in service of a successful integration of a new paradigm in which drugs and psychotherapy interact.

Considering the paradigmatic tensions between psychedelic research and biological psychiatry can help us understand the challenges faced by the FDA in judging MDMA-Assisted Therapy (FDA, 2023), and improve future regulatory processes. On June 4th 2024, the advisory committee voted that there is insufficient evidence for drug efficacy and that the benefits do not outweigh the risks. This was followed by a rejection by the FDA a couple of months later. While some of the critiques raised against MAPS/Lykos studies are valid, an important question to contemplate is whether the regulatory procedure poses the right frameworks, methods, and expertise required to regulate psychedelic-assisted therapy (PAT). I argue that this event represents paradigmatic tensions within psychopharmacology, which are important to consider when integrating psychedelics into the medical system.

Invoking Kuhn (1962/1997), the old paradigm through which we understand the psychopharmacological effects of drugs is biomedical, for example, drugs are thought to correct imbalances in the brain, and their therapeutic effects are context-independent. Even though many consider the biomedical paradigm outdated, it is still the primary scientific paradigm through which drugs are regulated. PAT is a strong anomaly challenging this old paradigm – it is a transformation-based medicine that does not require chronic administration, it is potentially transdiagnostic, it is context-dependent, and it leads to long-term clinical changes related to psychological processes influenced by the substance (Scheidegger, 2021; Schenberg, 2018).

In the ‘old’ *additive* paradigm, the therapeutic effects of drugs are added to context (drug + context). In the Kuhnian sense, this is the ‘normal’ science – the prevailing framework defining existing theories and methods. In the ‘new’ *interactive* paradigm, the therapeutic effects of drugs are *interactive* with context (drug X context) (Carhart-Harris et al., 2017; Pronovost-Morgan et al., 2023). ‘*New*’, in the Kuhnian sense, challenges the old one, as it offers different perspectives, methods, and theories that better explain the data and solve anomalies. It is not necessarily novel, and indeed the interactive model is already used in some sectors and practices within psychiatry. Furthermore, interactive proto-paradigms existed in the past and are accepted in the present by some research niches. Yet, they are far from being dominant.

In this ‘new’ paradigm, the intensity of the therapeutic experiences is dependent on contextual factors (Roseman et al., 2024). For example, the therapeutic alliance or the trust with the facilitators increases the chance of having beneficial therapeutic experiences in clinical trials (Levin et al., 2024; Murphy et al., 2022; Zeifman et al., 2024) and naturalistic studies (Kettner et al., 2021; Pontual et al., 2022). Furthermore, naturalistic studies show that a lack of psychological support can lead to extended difficulties after psychedelic use (Evans et al., 2023; Simonsson et al., 2023). This new science seeks to emerge from the *interaction* of psychopharmacology and psychotherapy, and such an interactive paradigm might also be relevant to other substances, such as Serotonin Reuptake Inhibitors (Branchi, 2011). Psychedelics are probably not unique in that sense, but they are the anomaly that challenges the status quo.

The most notable manifestation of the *additive* paradigm is that the regulatory process mainly relies on the blinded Randomized Controlled Trial (RCT) with two arms (drug and placebo). The blinded RCT supposes that context is noise to control for. Other standard study procedures recognised by the FDA – like comparative efficacy trials, dose-response studies, noninferiority studies, and withdrawal studies – do not take into consideration the interaction with contextual factors and hence also fall under the additive paradigm. It is argued that the introduction of this blinded RCT as the gold standard in the 1960s, due to the thalidomide scandal, was when psychedelic therapy halted and lost its ‘scientific’ credibility, as it could not prove itself through the blinded RCT (Oram, 2012). This represented a larger split between psychotherapy and psychopharmacology – disciplines that were once much closer to each other.

In the emerging new science, drug effects are interactive with context, requiring other study designs to explore this interaction. One potential design is a two-by-two design with drug/no-drug and psychotherapy/no-psychotherapy as the experimental arms (though some might argue that providing the drugs without the psychotherapy is unethical as it puts patients at risk).

From the perspective of the FDA advisory committee – who bear significant responsibility for bringing safe therapy to the market – the data was not good enough, and the risk seemed too high. The risks, efficacy, and liability are judged through the old paradigm, and it is plausible that they did not have the knowledge and frameworks to serve effectively as experts, and authorising something one cannot evaluate appropriately is risky. As the FDA does not regulate psychotherapy, most experts’ backgrounds were in psychiatry and medicine, with only one psychologist and one sociologist (FDA-Advisory-Committee, 2024).

Paradoxically, while the FDA does not regulate psychotherapy, most challenges in judging this procedure stem from the psychotherapeutic elements in MDMA-assisted therapy: functional unblinding is related to the intense emotional states that MDMA induces in a therapeutic context – such experiences are part of the psychotherapeutic procedure and trying to minimise them is contradictory to what is offered by PAT. Flagging the experience as a critical limitation – by pointing to the unavoidable functional unblinding – signals a wish for less ‘experience’. Such paradox is created by the necessity of judging PAT by methods created for a biomedical paradigm and the wish for this modality to align with those methods.

Furthermore, the most notable flagged ethical concerns were related to sexual violations taking place within the psychotherapeutic relationship – violations which can take place with other drugs as well, such as anaesthetics and sedatives. The fact that the FDA claimed that they do not regulate psychotherapy but at the same time did consider the risks embedded in the psychotherapeutic relation suggests we are facing a paradox; and paradoxes, as we know, are located on the edges of theoretical frameworks, where knowledge reaches its limits. Paradoxes hint at a shift that might take place.

The wishes to blind visionary states (pun intended) or assess the risk of therapeutic relations without regulating psychotherapy are comical, as they reveal an apparent bug in a regulatory system and expose how science can become a bureaucracy. Indeed, the problems of blinding are not unique to psychedelic research and appear in many other studies (Baethge et al., 2013; Laursen et al., 2023; Moncrieff et al., 1996). Psychedelics amplify the issues more clearly.

Nevertheless, it is crucial to note that some of the critiques towards MAPS might have been valid regardless of the paradigmatic framework: the psychotherapy could have been better manualised to standardise it for research; the enjoyable ‘adverse’ events such as euphoria – which are considered an abuse risk – should have been reported; sub-group analysis of MDMA-naïve versus MDMA-experienced subjects should have been presented; and most crucially MAPS/Lykos could have presented what measures were introduced to reduce violations of the therapeutic relationship after these took place in their trial. But even when considering other valid critiques, there seems to be an agreement that the ‘-assisted therapy’ component was the central tension point (Marks, 2024).

If the FDA seeks to address unmet needs, it must allow innovation to occur. When confronted with novelty, research methods should be adaptable. If psychotherapy is essential, regulators should determine how to involve psychotherapists’ expertise, who have long judged ethical and relational issues and evaluated the quality of different therapeutic frameworks. The FDA has previously shown signs of adapting to new science, and the advisory committee might not have followed previous flexibilities offered by the FDA. The FDA has various advisory committees and not all are strictly biological, like the Digital Health Advisory Committee. It might make sense to create a Psychotherapy with Drugs Advisory Committee to accommodate future applications with appreciation to the wisdom carried by an emergent research community – even if that wisdom has not been rigorously tested yet.

If regulators continue with the current status quo, we can expect two outcomes, which are already trending: first, the psychotherapeutic element will disappear, making the drugs less effective or not effective at all and potentially unsafe (Schenberg et al., 2024), as happened in the 60s (Oram, 2012). Providers will create a biomedical *façade* for their psychotherapeutic intervention, and the distance between clinical practitioners and researchers will increase. Therapists might continue to do psychotherapy and train other therapists on how to do so, while the researchers will downplay these elements when they communicate the results in medical journals and mainstream media. This will harm the truthfulness of the reported science – to please the regulator – and can compromise safety and efficacy (Schenberg et al., 2024). The second outcome, which is already taking place as well, is relegating psychedelic therapy to the realm of alternative medicine, with providers operating outside the medical establishment.

To increase accessibility beyond cultural milieus that have access to underground practices or retreats, the therapeutic application of psychedelics will require a paradigm shift in how we test and regulate these drugs. Psychedelic researchers will have to conduct research that aims to strengthen the *interactive* paradigm while following the rigour of the regulatory system but without assimilating to the current status quo. Furthermore, improved standardisation and fidelity assessments will help professionalise the introduction of psychotherapy into psychedelic clinical trials. Psychotherapy might be narrowed and simplified for the sake of the trials while being expanded on in the rollout. Regulators who understand these paradigmatic tensions must be forgiving and flexible towards those courageous enough to expand our frameworks – work with them instead of against them – and signal towards others that novelty and creativity are worth the pursuit.
